# Phylodynamic estimation of the within-host evolutionary rate of extended-spectrum beta-lactamase-producing Enterobacterales

**DOI:** 10.1099/mgen.0.001499

**Published:** 2025-09-05

**Authors:** Etthel M. Windels, Lisandra Aguilar-Bultet, Isabelle Vock, Laura Maurer Pekerman, Sarah Tschudin-Sutter, Tanja Stadler

**Affiliations:** 1 Department of Biosystems Science and Engineering, ETH Zurich, Basel, Switzerland; 2 Swiss Institute of Bioinformatics, Lausanne, Switzerland; 3 Swiss Tropical and Public Health Institute, Allschwil, Switzerland; 4 Division of Infectious Diseases, University Hospital Basel, University of Basel, Basel, Switzerland; 5 Department of Clinical Research, University Hospital Basel, University of Basel, Basel, Switzerland; 6 University Children's Hospital Basel, Basel, Switzerland

**Keywords:** extended-spectrum beta-lactamase-producing Enterobacterales (ESBL-PE), phylodynamics, within-host evolution

## Abstract

Despite their clinical relevance, the within-host evolution of extended-spectrum beta-lactamase (ESBL)-producing Enterobacterales is still poorly understood. To estimate the within-host evolutionary rates of ESBL-producing *Escherichia coli* and *Klebsiella pneumoniae* species complex, we fitted phylodynamic models to genomic sequence data of longitudinally collected rectal swabs from 63 colonized hospital patients. We estimated an average within-host evolutionary rate of 7.71e-07 [4.60e-07,1.10e-06] mutations/site/year for *E. coli* and 4.20e-07 [1.57e-07,7.14e-07] mutations/site/year for the *K. pneumoniae* species complex, with limited variation observed across patients and sequence types. These estimates are presumably the most accurate reported so far and are useful for future epidemiological and evolutionary studies.

**Impact Statement**

Understanding the within-patient evolutionary rates of extended-spectrum beta-lactamase (ESBL)-producing *Escherichia coli* and *Klebsiella pneumoniae* species complex is critical for elucidating their evolutionary dynamics and transmission potential. In this study, we employed Bayesian phylodynamic models to analyse longitudinal isolates collected over a 10-year period from hospitalized patients. Our findings reveal limited variability in evolutionary rates across patients and sequence types, suggesting evolutionary stability within these pathogens in hospital settings. This work provides new insights into the persistence and evolutionary dynamics of ESBL-producing Enterobacterales, offering valuable guidance for antimicrobial resistance surveillance and infection prevention strategies.

## Data Summary

The data used in this study are publicly available in the NCBI database under the BioProject number PRJNA910977. Supporting metadata are provided in Supplementary Material 1. The alignments and code for the phylodynamic analyses, including the BEAST2 XML files, are available at https://github.com/EtthelWindels/esbl-pe_mutation.

## Introduction

Extended-spectrum beta-lactamase-producing Enterobacterales (ESBL-PE) are clinically significant micro-organisms associated with increased morbidity, mortality and healthcare costs due to their resistance to a wide range of beta-lactam antibiotics [1,2]. Understanding their transmission dynamics, particularly over long time periods, is critical to mitigate their impact. To investigate potential transmission events, it is essential to account for the diversity and evolution of pathogens within an individual host. In hospital settings, sequences from strains collected at different time points and body sites are valuable for such investigations. In a previous study [3], we demonstrated the long persistence and very low genomic variation of ESBL-PE strains within patients. We estimated preliminary within-host evolutionary rates by comparing the number of SNPs between consecutive isolates and the time between samplings. However, this method did not account for non-parsimonious mutation histories (including back mutations, multiple mutations at the same site and parallel mutations). Moreover, phylogenetic relationships were ignored, thereby potentially underestimating the true divergence times and hence overestimating the evolutionary rates. In this study, we aimed to overcome these limitations by using Bayesian phylodynamic models, which consider the phylogenetic tree as well as an evolutionary model accounting for unobserved mutations, to obtain more accurate estimates of within-patient evolutionary rates for ESBL-producing *Escherichia coli* and *Klebsiella pneumoniae* species complex collected during a 10-year longitudinal study.

## Methods

### Study population

We used previously sequenced serial isolates from an observational cohort study at the University Hospital Basel, Switzerland [3]. Patients included in this study were admitted to the hospital between January 2008 and December 2018 and had ESBL-PE belonging to the same species (*E. coli* or the *K. pneumoniae* species complex) detected in at least two consecutive rectal swabs. In total, 185 rectal swab isolates were collected, comprising 134 *E. coli* isolates from 47 patients and 55 *K*. *pneumoniae* species complex isolates from 19 patients. Two patients (patients 17 and 18) harboured both species. The isolation procedure is described in the Supplementary Methodology (Supplementary Material 2). The isolates included in this study along with their associated patients, patient metadata, sequence type (ST) classification and cluster assignments (i.e. isolates within the same cluster belong to the same strain) are summarized in Supplementary Material 1.

### Multiple sequence alignments

Raw Illumina sequencing reads were processed as described before [3]. Per-patient alignments were generated using Snippy v.4.6.0 (https://github.com/tseemann/snippy) (Supplementary Methodology). A patient-specific reference was generated by concatenating all contigs from the first sample of each patient. For patients harbouring two different strains, two different alignments and references were generated. The query samples were taken from the consensus of each pairwise mapping and were concatenated in the same way. From the complete alignments (4,714,797 sites for *E. coli*; 5,654,681 sites for the *K. pneumoniae* species complex), only the variable sites were kept. Only patients for whom at least three serial isolates belonging to the same strain were available were retained for the main analyses (Supplementary Material 1). STs were identified using Ridom SeqSphere+ v.6.0 (Ridom, Münster, Germany), and only STs with at least three patients, of which at least one patient had at least three serial isolates, were retained for the main analyses (Supplementary Material 1).

### Phylodynamic model

A constant population size coalescent model was fitted onto the set of per-patient sequence alignments, with patient-specific parameters for the effective population size. We set the non-informative Jeffreys prior on the effective population size (Table S1, available in the online Supplementary Material), as limited knowledge is available on this parameter. We further assumed a strict molecular clock and a general time-reversible nucleotide substitution model with four gamma rate categories to account for site-to-site rate heterogeneity (GTR+Γ_4_). All substitution model parameters were assumed to be shared across all patients, which facilitated the calibration of near-ultrametric trees. In a first analysis, the evolutionary rate was also assumed to be shared across all patients. In the follow-up analyses, the model was additionally parametrized with either patient-specific or ST-specific evolutionary rate multipliers. The overall patient-specific or ST-specific evolutionary rate was then equal to the shared evolutionary rate multiplied by the respective multiplier. All parameters and their prior distributions are listed in Table S1.

### Phylodynamic inference

We performed phylodynamic inference using the feast package v8.3.1 (https://github.com/tgvaughan/feast/releases/tag/v8.3.1) in BEAST v2.7.6 [4,5]. Data from each species (*E. coli* and *K. pneumoniae* species complex) were analysed independently. Variable SNP alignments were augmented with a count of invariant A, C, G and T nucleotides [6]. For each analysis, three independent Markov Chain Monte Carlo chains were run, with states and trees sampled every 100,000 steps. Convergence was assessed with Tracer [7], confirming that the effective sample size was at least 200 for the parameters of interest. Ten per cent of each chain was discarded as burn-in, and the remaining samples across the three chains were pooled with LogCombiner [5], resulting in at least 50,000,000,000 iterations in combined chains.

### Sensitivity analyses

The robustness of the phylodynamic inference to prior assumptions was tested by setting a Lognormal(−14.60,1.25) prior (corresponding to a mean in real space of 10e-6) on the evolutionary rate, as well as by using an exponential growth coalescent model with patient-specific growth rate parameters and a Laplace(0.001,0.5) prior distribution on these growth rates. Moreover, we tested the effect of including patients for which only two isolates are available. Finally, instead of estimating a shared evolutionary rate and patient-specific multipliers, we tested the performance of a model with independent patient-specific evolutionary rate parameters, with the same Lognormal(−13.82,1.25) prior distribution set on each evolutionary rate.

## Results

### Estimation of average within-patient evolutionary rates

**Fig. 1.**
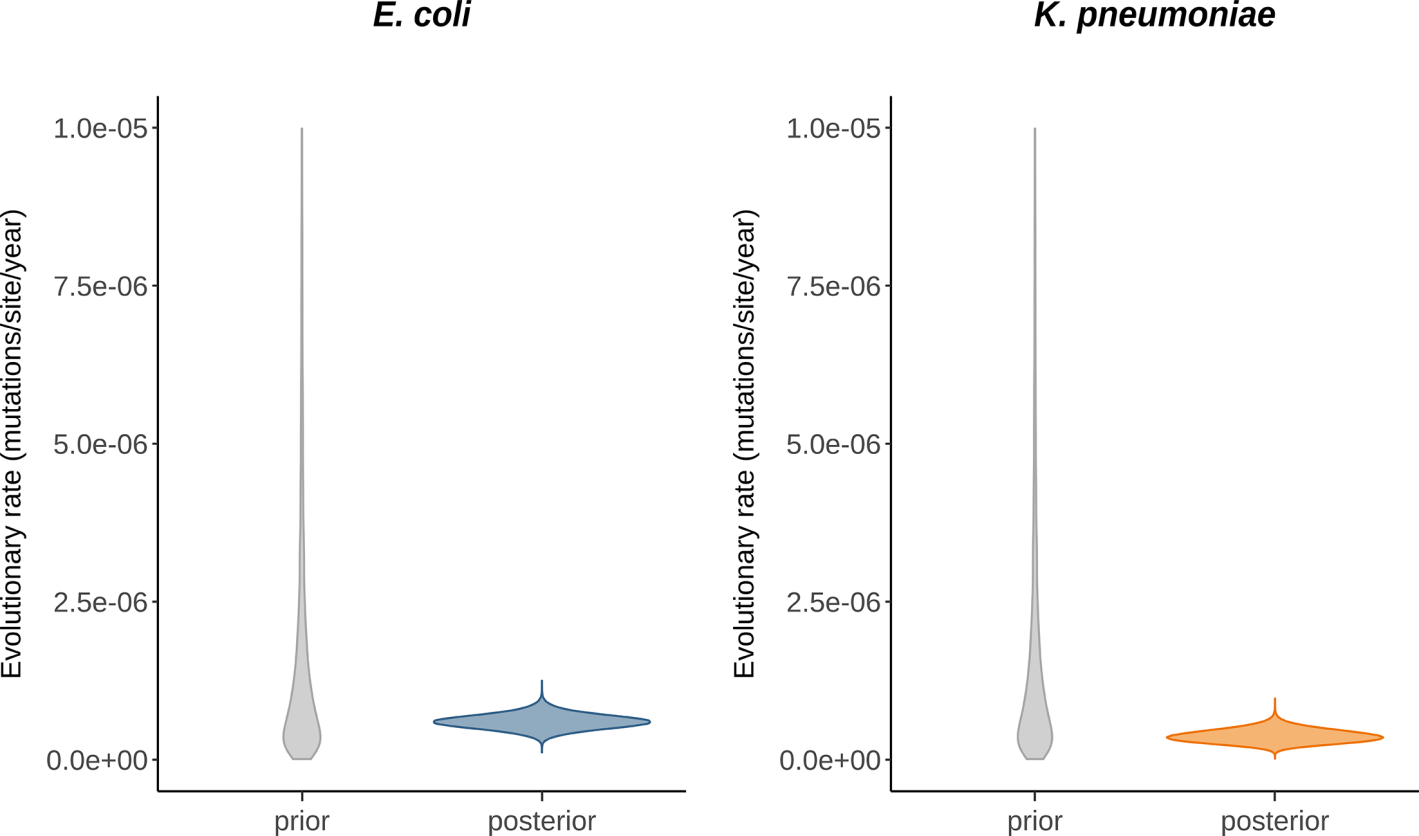
Average within-patient evolutionary rate estimates. Prior (grey) and posterior (coloured) distributions of the within-patient evolutionary rate, averaged over all patients for which at least three serial isolates were available (21 patients with 78 isolates for *E. coli* and 7 patients with 29 isolates for the *K. pneumoniae* species complex). The posterior mean and 95% HPDI correspond to 6.04e-07 [3.69e-07,8.40e-07] mutations/site/year for *E. coli* and 3.75e-07 [1.77e-07,5.83e-07] mutations/site/year for the *K. pneumoniae* species complex.

In a previous study at the University Hospital Basel, Switzerland, 185 rectal swab isolates were longitudinally collected from 63 patients colonized with ESBL-PE [3]. We used these data to generate per-patient sequence alignments and fitted a constant population coalescent model with a strict clock, assuming a shared within-patient evolutionary rate for all patients per species. Only patients with at least three serial isolates belonging to the same strain (28 out of 63 patients and 107 out of 185 isolates) were included in the analysis. Only nucleotide substitutions were considered as mutations, while insertions and deletions were ignored. Importantly, our approach did not capture highly deleterious or lethal mutations, indicating that the estimated evolutionary rates reflect only those mutations that are observable. Inference of these rates was done in a Bayesian setting, meaning that we inferred posterior distributions which reflect the information in the data about each parameter, in combination with the prior distribution. The posterior mean for the average evolutionary rate was estimated to be 6.04e-07 (95% highest posterior density interval, HPDI: [3.69e-07,8.40e-07]) mutations/site/year for *E. coli* and 3.75e-07 (95% HPDI: [1.77e-07,5.83e-07]) mutations/site/year for the *K. pneumoniae* species complex (Fig. 1). These estimates are slightly lower than the estimates reported before (1.4e-06 (interquartile range, IQR: [7.6e-07,3.2e-06]) mutations/site/year for *E. coli* and 1.5e-06 (IQR: [7.3e-07,3.5e-06]) mutations/site/year for the *K. pneumoniae* species complex) [3]. When all patients – including those with only two serial isolates – were considered, the posterior estimates increased slightly (8.69e-07 [6.21e-07,1.09e-06] mutations/site/year for *E. coli* and 4.70e-07 [2.41e-07,7.14e-07] mutations/site/year for the *K. pneumoniae* species complex), and the posterior distributions more closely followed the prior (Fig. S1), suggesting that the isolates of these patients are uninformative about the evolutionary rate and may even slightly bias the posterior estimates in combination with the chosen prior.

### Estimation of patient-specific within-patient evolutionary rates

**Fig. 2.**
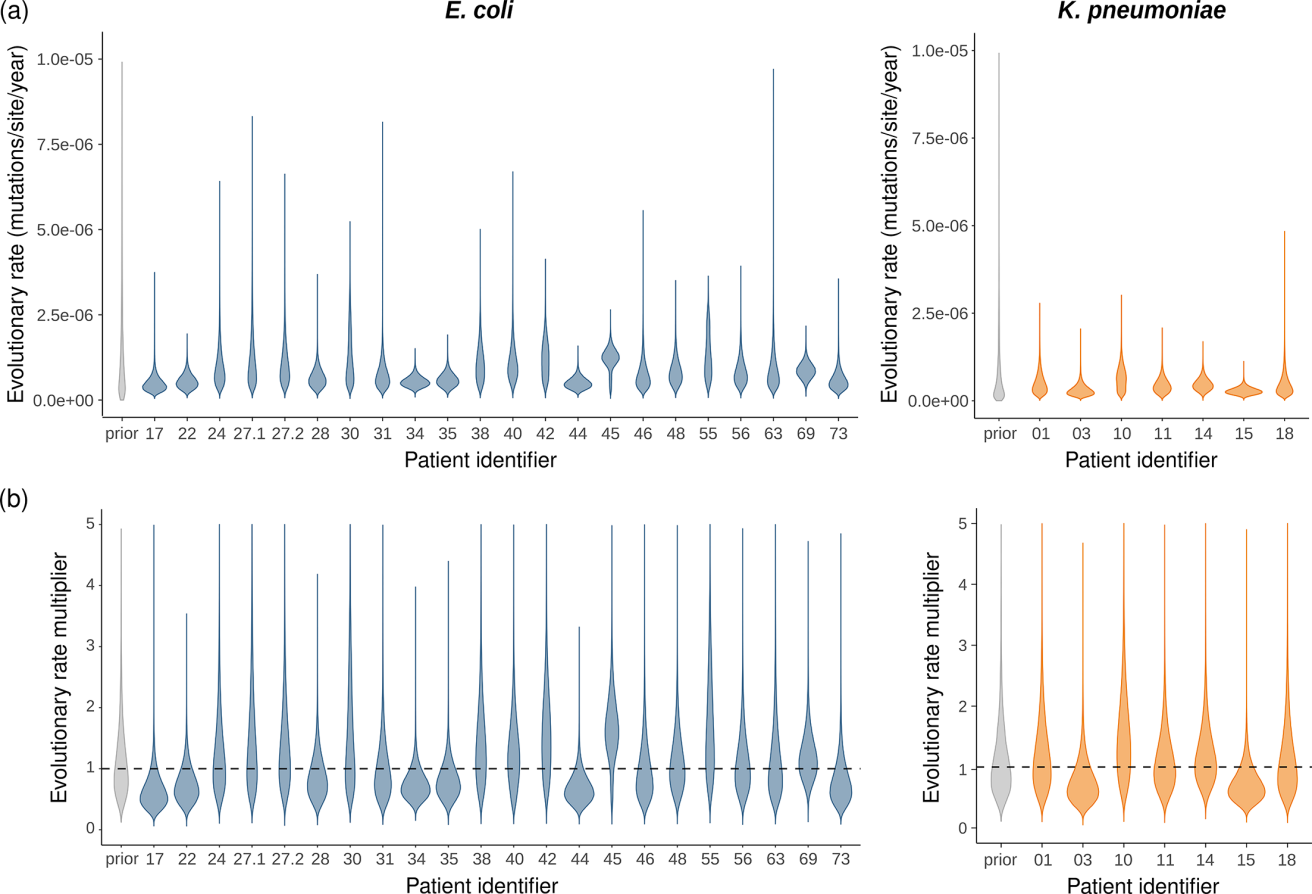
Patient-specific within-patient evolutionary rate estimates. (**a**) Prior (grey) and posterior (coloured) distributions of patient-specific within-patient evolutionary rates, estimated for all patients for which at least three serial isolates were available (21 patients with 78 isolates for *E. coli* and 7 patients with 29 isolates for the *K. pneumoniae* species complex). Each patient-specific evolutionary rate estimate corresponds to the product of the average evolutionary rate estimate (7.71e-07 [4.60e-07,1.10e-06] mutations/site/year for *E. coli* and 4.20e-07 [1.57e-07,7.14e-07] mutations/site/year for the *K. pneumoniae* species complex) and a patient-specific multiplier estimate. (**b**) Prior (grey) and posterior (coloured) distributions of patient-specific evolutionary rate multipliers. The posterior estimates are close to one for most patients, suggesting limited patient-to-patient variability. Patient identifiers 27.1 and 27.2 correspond to the same patient but different strains, so two evolutionary rates were estimated for this patient.

We then adjusted the phylodynamic model to infer a within-host evolutionary rate per patient. In addition to the average evolutionary rate informed by all patient alignments, this model was further parametrized with patient-specific evolutionary rate multipliers, with each patient-specific evolutionary rate corresponding to the product of the average evolutionary rate and the patient-specific multiplier. The phylogenetic trees inferred under this model are shown in Fig. S2. The resulting estimates for the average evolutionary rate were similar to the estimates from the previous analysis (7.71e-07 (95% HPDI: [4.60e-07,1.10e-06]) mutations/site/year for *E. coli* and 4.20e-07 (95% HPDI: [1.57e-07,7.14e-07]) mutations/site/year for the *K. pneumoniae* species complex). The posterior estimates for the patient-specific multipliers were close to one for most patients, suggesting limited patient-to-patient variability in within-host evolutionary rates (Fig. 2). When additionally considering patients for which only two isolates were available, the estimates for the average evolutionary rate again increased slightly. Consequently, the patient-specific evolutionary rate for some patients was estimated to be lower than this average (Fig. S3).

We tested the robustness of our estimates to prior assumptions, by setting a different prior distribution on the average evolutionary rate (Fig. S4) and by assuming an exponential growth coalescent model (Fig. S5). In both cases, the evolutionary rate estimates were almost unaffected. When patient-specific evolutionary rates were estimated independently instead of through a shared evolutionary rate combined with independent multipliers, the overall trends remained the same, although with increased uncertainty in the posterior estimates (Fig. S6).

### Estimation of ST-specific within-patient evolutionary rates

**Fig. 3.**
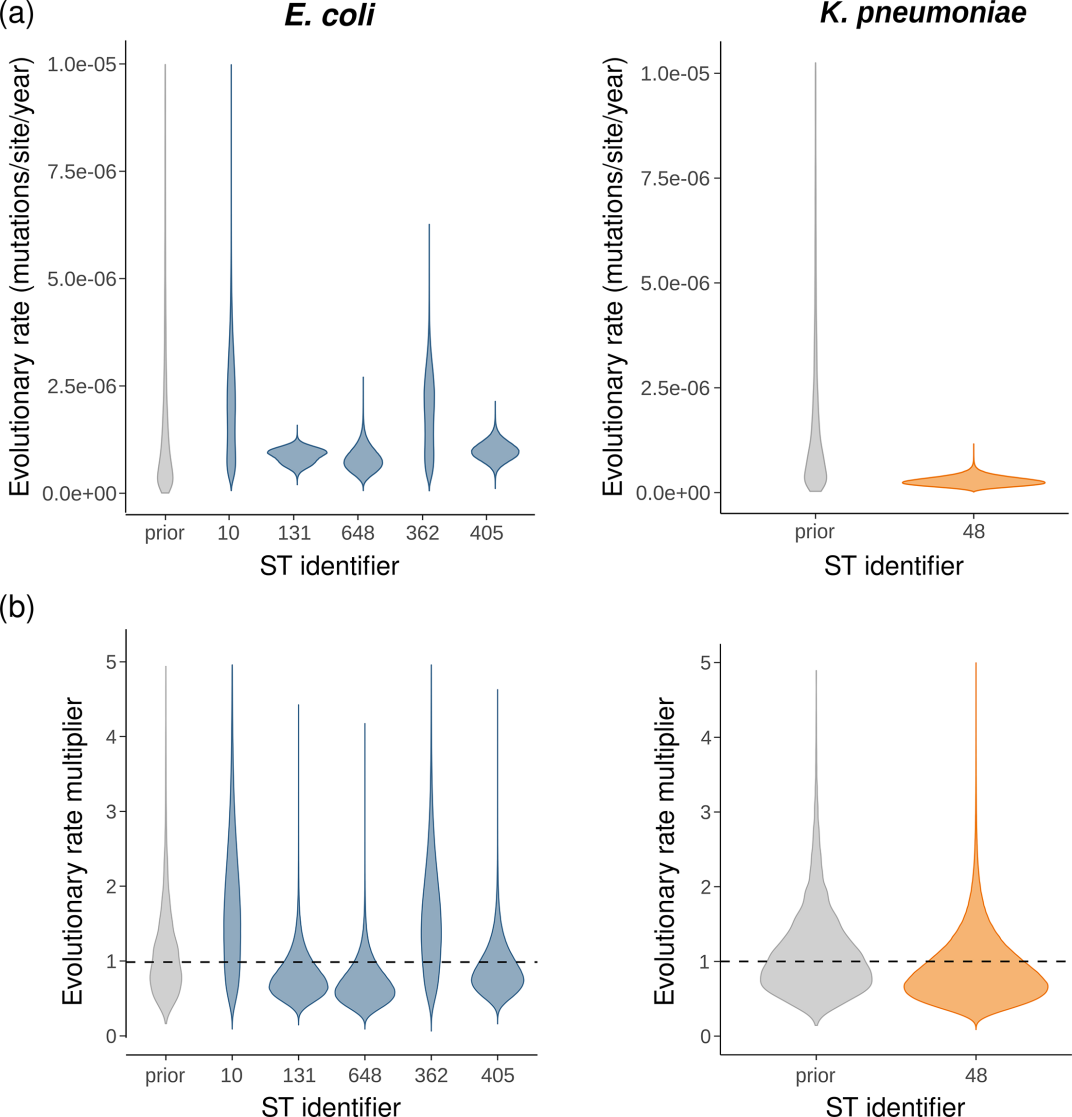
ST-specific within-patient evolutionary rate estimates. (**a**) Prior (grey) and posterior (coloured) distributions of ST-specific within-patient evolutionary rates, estimated for all STs with at least three patients, of which at least one patient with at least three serial isolates (30 patients with 87 isolates belonging to 5 STs for *E. coli* and 3 patients with 9 isolates belonging to 1 ST for the *K. pneumoniae* species complex; Table S2). Each ST-specific evolutionary rate estimate corresponds to the product of the average evolutionary rate estimate (1.17e-06 [4.73e-07,1.94e-06] mutations/site/year for *E. coli* and 3.40e-07 [3.97e-08,7.65e-07] mutations/site/year for the *K. pneumoniae* species complex) and an ST-specific multiplier estimate. (**b**) Prior (grey) and posterior (coloured) distributions of ST-specific evolutionary rate multipliers. The posterior estimates are close to one for most STs, implying that the data do not support an association between ST and evolutionary rate.

STs are unique identifiers assigned to bacterial strains based on the allelic profile of multiple housekeeping genes. To check for an association between STs and within-patient evolutionary rates, we inferred an evolutionary rate for each ST with at least three sampled patients, of which at least one patient had at least three serial isolates (Table S2). Similar to the patient-specific evolutionary rate inference, we generated per-ST alignments and inferred an average evolutionary rate informed by all ST alignments, as well as ST-specific evolutionary rate multipliers. The average evolutionary rate estimate for *E. coli* (1.17e-06 (95% HPDI: [4.73e-07,1.94e-06]) mutations/site/year) was slightly higher than in previous analyses, while the estimate for the *K. pneumoniae* species complex (3.40e-07 (95% HPDI: [3.97e-08,7.65e-07]) mutations/site/year) was similar (Fig. 3). However, the patient set used in this analysis is slightly different from the one used in previous analyses, and the number of patients per ST varies, making it difficult to predict the effect of clustering by ST on the average evolutionary rate estimate. For the *K. pneumoniae* species complex, only one ST fulfilled the inclusion criteria, making the multiplier estimate redundant. For *E. coli*, the ST-specific evolutionary rate multiplier estimates did not deviate from one and showed limited variability, implying that the data do not support an association between the within-patient evolutionary rate and ST. Including all patients in the analysis resulted in a slight increase in the estimated average evolutionary rate for the *K. pneumoniae* species complex, and again limited ST-to-ST variability (Fig. S7).

## Discussion

In this study, we used phylodynamic models to estimate the within-patient evolutionary rate of ESBL-producing *E. coli* and *K. pneumoniae* species complex from longitudinal isolates collected in a hospital setting. This evolutionary rate refers to the rate at which new mutations – excluding highly deleterious and lethal ones – arise within host, as opposed to substitution rates which indicate the rate at which mutations are fixed in a population. Our study was exclusively focused on within-host evolutionary dynamics; evolutionary rates between hosts may differ due to distinct selective pressures and were beyond the scope of this work. The within-host evolutionary rates were estimated as an average over all patients, as well as on a per-patient and per-ST basis, using models that were parametrized such that the information in the available data was used as efficiently as possible. The resulting estimates of the average within-patient evolutionary rate were slightly lower than reported previously [3]. These previous estimates were obtained from the same set of longitudinal isolates (including the additional patients with only two isolates), by calculating the number of observed SNPs divided by the time between samplings, specifically comparing each isolate to the first one collected from the same patient and taking the average over all pairs. However, this approach considers only the sampling interval as a proxy for evolutionary time, which can underestimate the actual time of divergence since the most recent common ancestor of all isolates in a patient. This is because different bacterial strains can diverge within the host long before they are sampled; therefore, the time between samplings does not necessarily reflect the real evolutionary timeline. This may partly explain the higher evolutionary rate estimates obtained in the previous study [3]. Additionally, the inclusion of patients with only two isolates in the previous study may have contributed to differences in estimates. Another difference is that our phylodynamic models include a substitution model accounting for unobserved mutations such as back mutations. If many such mutations occurred, ignoring them could result in an overestimation of the evolutionary rate. For *E. coli*, the new estimates derived in this study correspond well to the previously reported within-host evolutionary rate of the non-pathogenic *E. coli* ED1a clone (6.90e-7 mutations/site/year) estimated from a single healthy individual using a similar Bayesian method [8].

The limited variation observed across patients was not entirely surprising, given the similar backgrounds of the majority of study participants in terms of medical history and previous comorbidities. The limited across-ST variation suggests that differences in adaptive potential are not directly caused by differences in evolutionary rate, not even for the globally spread ST131 of *E. coli*.

The per-patient evolutionary rate was estimated in two different ways, either by estimating the evolutionary rate independently for each patient alignment (with the same, relatively broad prior distribution set on each individual evolutionary rate) or by combining all alignments to inform a shared evolutionary rate (on which a broad prior was set) and including patient-specific multipliers (with a narrow prior around one) in the model to allow for patient-to-patient variation. The second method resulted in lower posterior uncertainty, likely because it uses the available data more efficiently by combining information from multiple patients while allowing for patient differences. Such analyses demonstrate the flexibility of Bayesian models, which is especially beneficial for small datasets.

A limitation of this study was that only a few isolates were available for most patients, resulting in rather large posterior uncertainty in patient-specific estimates. Our analyses showed that patients with less than three isolates are better excluded, as they result in more noisy and potentially skewed estimates given our prior choices. Future studies including more patients, and particularly more isolates per patient, have the potential to further refine the within-host evolutionary rates reported here.

## Supplementary material

10.1099/mgen.0.001499 Supplementary Material 1.

10.1099/mgen.0.001499 Supplementary Material 2.

## Abbreviations

ESBL: extended-spectrum beta-lactamase
ESBL-PE: extended-spectrum beta-lactamase-producing Enterobacterales
HPDI: highest posterior density interval
IQR: interquartile range
ST: sequence type
