# Supplementary material for: Phylodynamic estimation of the within-host evolutionary rate of extended-spectrum beta-lactamase-producing Enterobacterales
Source: Microb Genom. 2025 Sep 5;11(9):001499. doi: 10.1099/mgen.0.001499 (PMC12413297; doi:10.1099/mgen.0.001499)
Supplement: Supplementary Material 2. [file mgen-11-01499-s002.pdf]

Supplementary Materials for

Phylodynamic estimation of the within-host evolutionary rate of extended-spectrum beta-lactamase-producing Enterobacterales

Etthel M. Windels[1,2,3]*, Lisandra Aguilar-Bultet[4,5]*, Isabelle Vock[4,5], Laura Maurer Pekerman[4,5,6], Sarah Tschudin-Sutter[4,5], Tanja Stadler[1,2#]

* These authors contributed equally to this work as first authors

[1] Department of Biosystems Science and Engineering, ETH Zurich, Basel, Switzerland
[2] Swiss Institute of Bioinformatics, Lausanne, Switzerland
[3] Swiss Tropical and Public Health Institute, Allschwil, Switzerland
[4] Division of Infectious Diseases, University Hospital Basel, University of Basel, Basel, Switzerland
[5] Department of Clinical Research, University Hospital Basel, University of Basel, Basel, Switzerland
[6] University Children's Hospital Basel, Basel, Switzerland

[#] corresponding author:
Prof. Tanja Stadler
tanja.stadler@bsse.ethz.ch

Supplementary Methodology

Sample preparation and post-sequencing quality control

As described in our recent publication (1), standard culture methods according to the Clinical and Laboratory Standards Institute (CLSI) guidelines (https://clsi.org/) were followed. During the isolation process, individual colonies with distinct morphology and color grown on Chromogenic screening agar plates (chromID ESBL, bioMérieux, Marcy-l'Étoile, France) were selected.

Subsequently, clonal cultures were obtained from those single colonies (after ESBL confirmation) and used for whole-genome DNA extraction and sequencing, to avoid that mixed bacterial populations were present in the same isolate.

Following whole-genome sequencing, we confirmed species identity using Kraken2 v.2.0.8 (2) and rMLST (https://pubmlst.org/species-id), and also excluded contaminated and low-quality genomes based on assembly size, contig number, etc. Additionally, during the genome submission to the National Center for Biotechnology Information (NCBI), the quality of each assembly was independently assessed by the curators with the CheckM tool (3) which further verified genome completeness and contamination.

Variant analyses per patient

Per-patient alignments were generated using Snippy v.4.6.0 (https://github.com/tseemann/snippy), with default parameters (minimum read depth of 10, minimum variant allele depth of 10, minimum variant allele frequency of 0.9, minimum based quality of 13 (PHRED), minimum mapping quality of 60 (MAPQ), and minimum variant quality score of 100). ClonalFrameML v.1.12 (4) was used to identify recombination sites.

All contigs from the first sample of each patient were concatenated to create a patient-specific reference. The query samples were taken from the consensus of each pairwise mapping and were concatenated in the same way. We used consensus sequences with a minimum variant allele frequency of 90%.

Three *E. coli* patients (patients 27, 30 and 48) harbored two different strains; therefore two different alignments and references were generated.

Supplementary Tables

Table S1: Prior distributions for the parameters of the phylodynamic model (see Materials and Methods for more information about the individual parameters)

Parameter	Prior
Effective population size	1/X
Shared evolutionary rate	Lognormal(-13.82,1.25)
Evolutionary rate multiplier	Lognormal(0,0.5)
Gamma shape	Exp(1)
AC substitution rate	Gamma(0.05,10)
AG substitution rate	Gamma(0.05,20)
AT substitution rate	Gamma(0.05,10)
CG substitution rate	Gamma(0.05,10)
GT substitution rate	Gamma(0.05,10)
Proportion of invariant sites	Uniform(0,1)

Table S2: Sequence types (STs) and corresponding patients used in the main analyses. Only STs with at least three patients, of which at least one patient had at least three serial isolates, were included. Patient identifiers 30.1 and 30.2 correspond to the same patient but different strains.

Species	ST	Patient identifiers
E. coli	10	18, 61, 63
E. coli	131	21, 24, 26, 27, 28, 29, 32, 34, 35, 38, 39, 40, 41, 43, 44, 45, 62, 66, 71
E. coli	362	30.1, 30.2, 60
E. coli	405	46, 52, 69
E. coli	648	22, 51, 55
K. pneumoniae species complex	48	08, 15, 17

Supplementary Figures

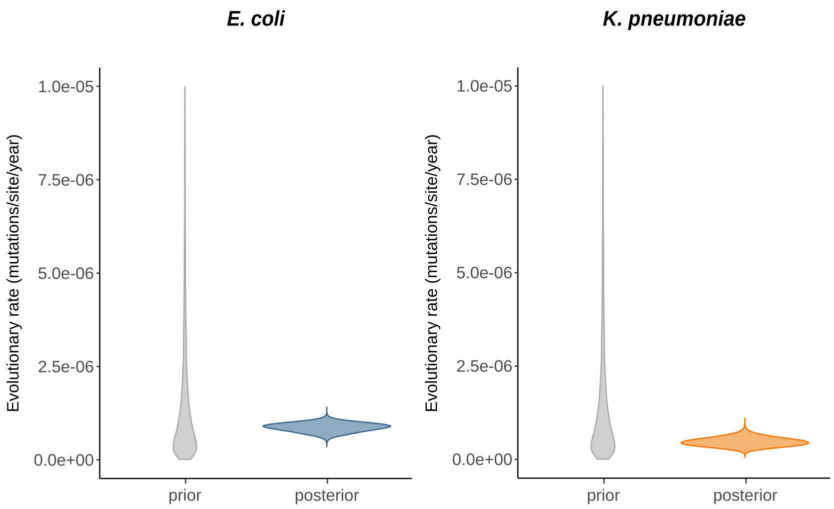

Figure S1: Average within-patient evolutionary rate estimates on all patients. Prior and posterior distributions of the within-patient evolutionary rate, averaged over all patients (including those with only two serial isolates). The posterior mean and 95% HPDI correspond to 8.69e-07 [6.21e-07,1.09e-06] mutations/site/year for *E. coli* and 4.70e-07 [2.41e-07,7.14e-07] mutations/site/year for *K. pneumoniae* species complex.

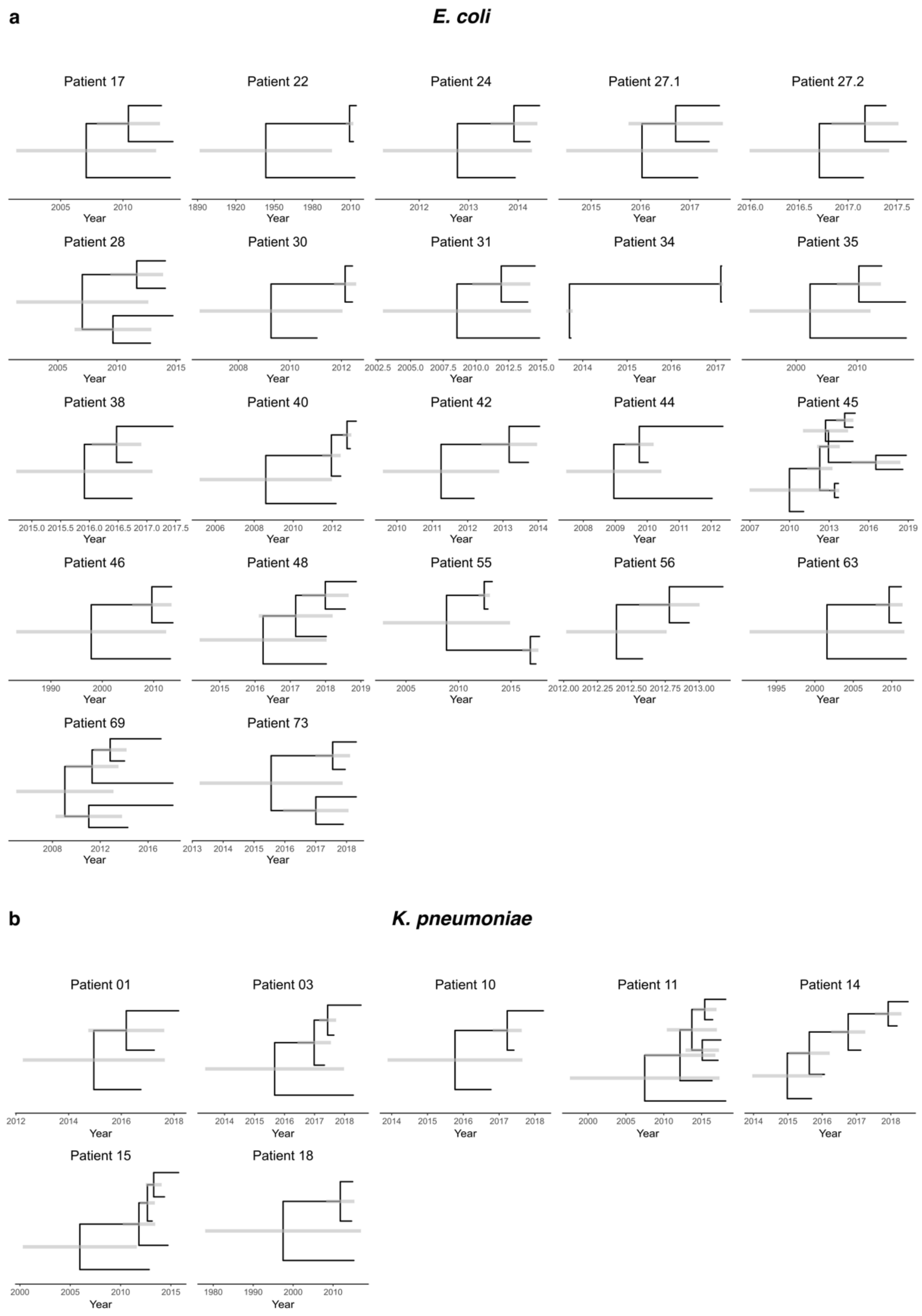

Figure S2: Posterior maximum clade credibility trees. Posterior maximum clade credibility trees per species and per patient, summarizing the posterior tree distribution resulting from the phylodynamic analyses on all patients for which at least three *E. coli* (a) or *K. pneumoniae* species complex (b) isolates were available. Grey shades represent 95% HPDIs on the node heights. Patient identifiers 27.1 and 27.2 correspond to the same patient but different strains.

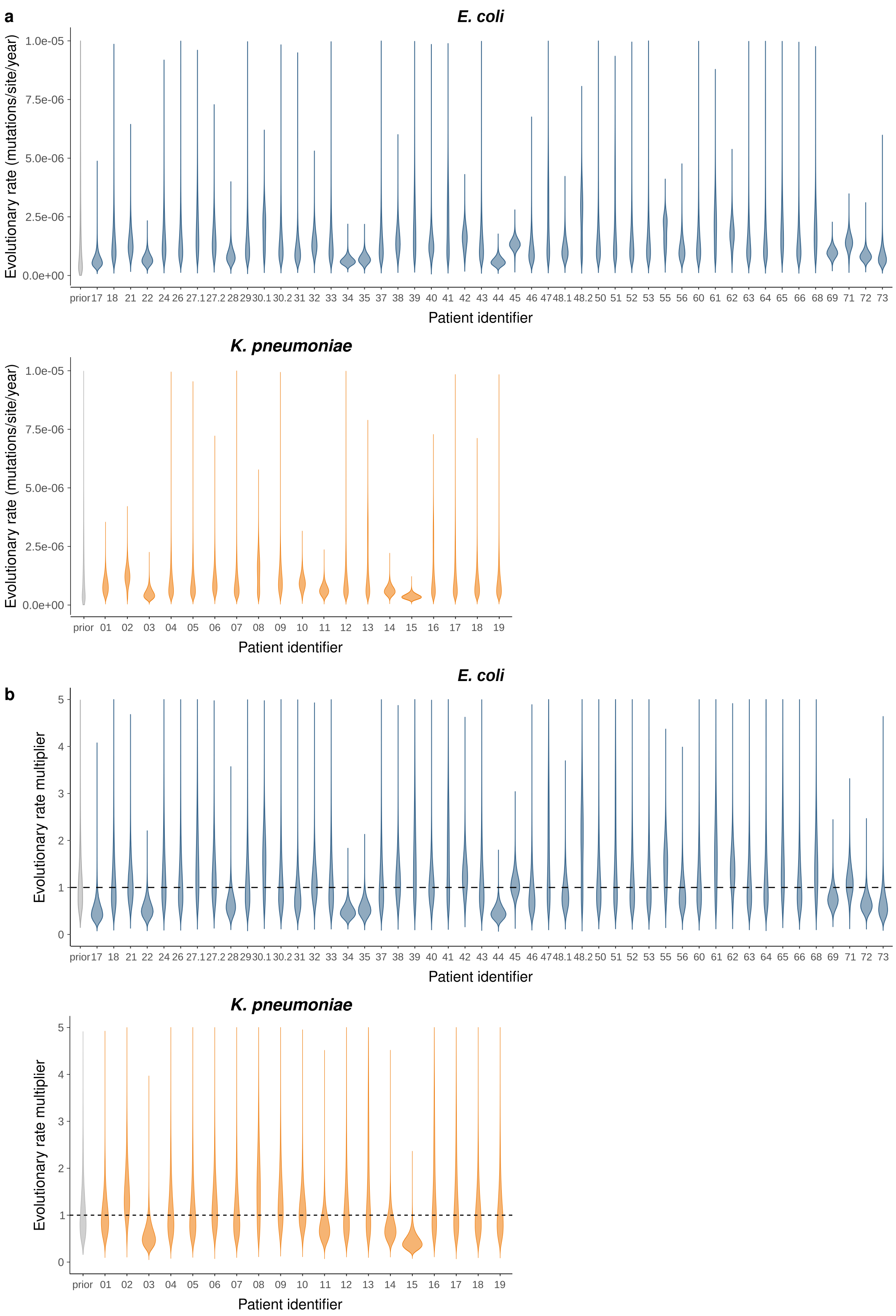

Figure S3: Patient-specific within-patient evolutionary rate estimates on all patients. a) Prior (grey) and posterior (colored) distributions of patient-specific within-patient evolutionary rates, estimated for all patients (including those with only two serial isolates). Each patient-specific evolutionary rate estimate corresponds to the product of the average evolutionary rate estimate

(1.28e-06 [8.72e-07,1.69e-06] mutations/site/year for *E. coli* and 8.69e-07 [3.85e-07,1.40e-06] mutations/site/year for *K. pneumoniae* species complex) and a patient-specific multiplier estimate. b) Prior (grey) and posterior (colored) distributions of patient-specific evolutionary rate multipliers. Patient identifiers 27.1/27.2, 30.1/30.2, and 48.1/48.2 correspond to the same patient but different strains, so two evolutionary rates were estimated for these patients. 30.1 and 48.1 correspond to the strains included in the main analyses. Patients 17 and 18 harbored both *E. coli* and *K. pneumoniae*.

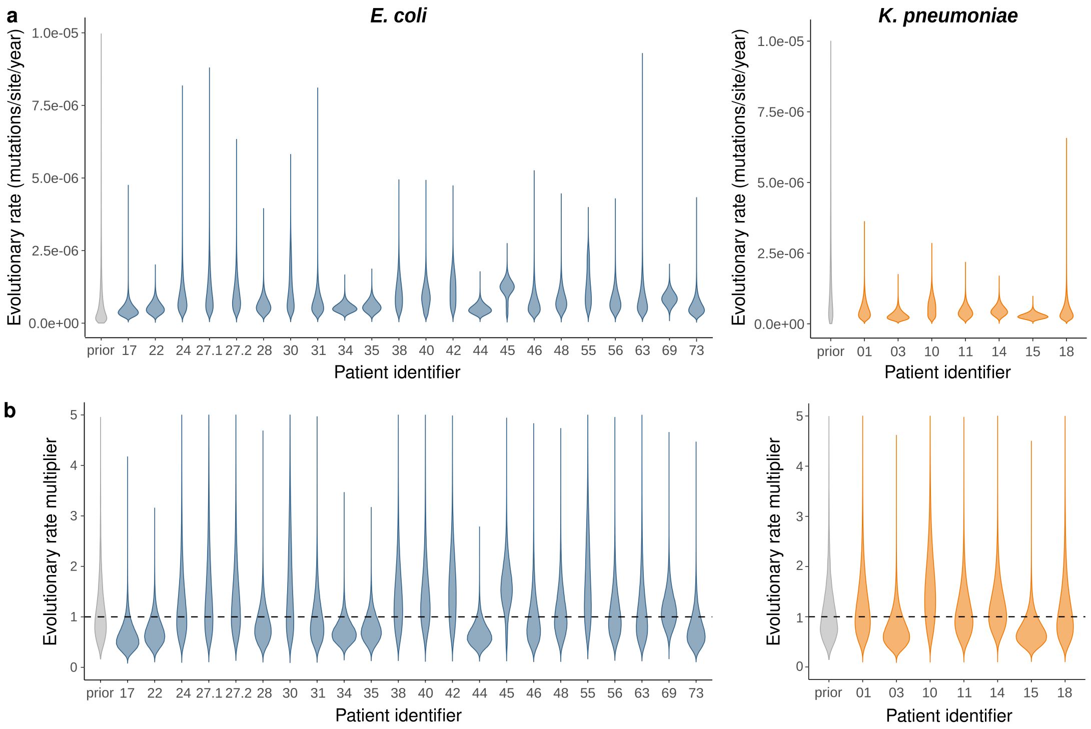

Figure S4: Patient-specific within-patient evolutionary rate estimates, using a different evolutionary rate prior. a) Prior (grey) and posterior (colored) distributions of patient-specific within-patient evolutionary rates, estimated for all patients for which at least three serial isolates were available, assuming a different prior distribution on the evolutionary rate (see Materials and Methods) as a sensitivity check. Each patient-specific evolutionary rate estimate corresponds to the product of the average evolutionary rate estimate (7.38e-07 [4.29e-07,1.07e-06] mutations/site/year for *E. coli* and 4.07e-07 [1.52e-07,6.95e-07] mutations/site/year for *K. pneumoniae* species complex) and a patient-specific multiplier estimate. b) Prior (grey) and posterior (colored) distributions of patient-specific evolutionary rate multipliers. All posterior distributions are close to those inferred under the main model (Figure 2), suggesting robustness to the choice of prior. Patient identifiers 27.1 and 27.2 correspond to the same patient but different strains, so two evolutionary rates were estimated for this patient.

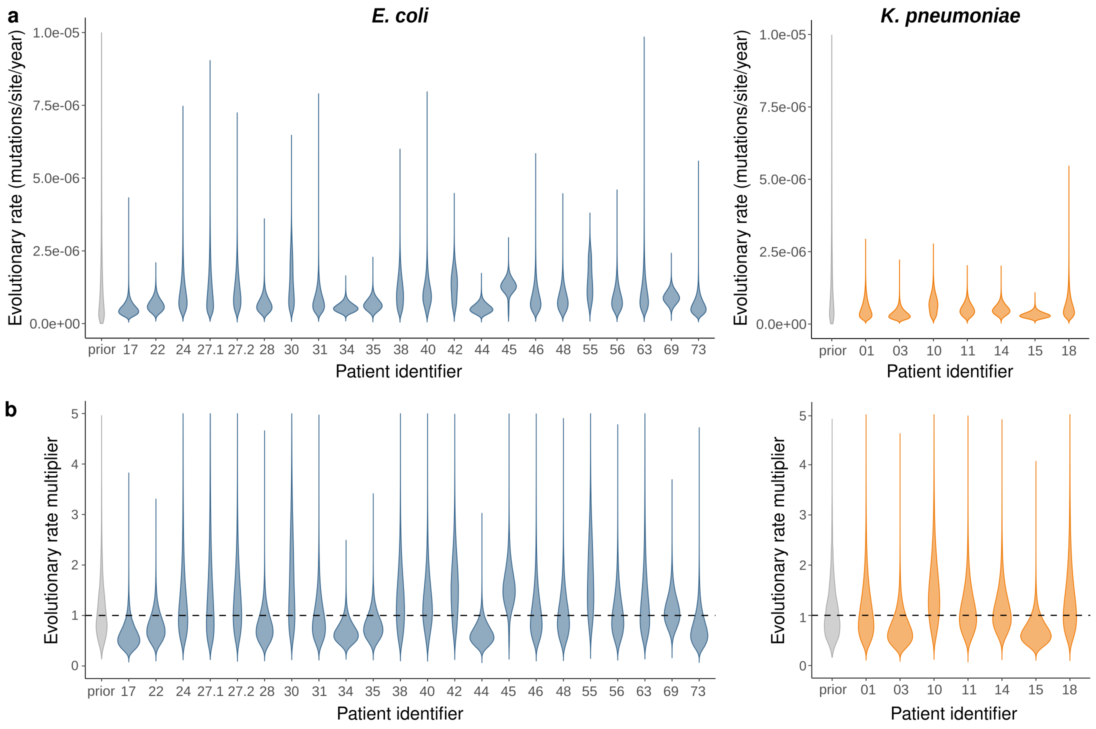

Figure S5: Patient-specific within-patient evolutionary rate estimates, using an exponential growth coalescent model. a) Prior (grey) and posterior (colored) distributions of patient-specific within-patient evolutionary rates, estimated for all patients for which at least three serial isolates were available, using an exponential growth coalescent model as a sensitivity check. Each patient-specific evolutionary rate estimate corresponds to the product of the average evolutionary rate estimate (8.39e-07 [5.28e-07,1.16e-06] mutations/site/year for *E. coli* and 4.76e-07 [2.05e-07,7.81e-07] mutations/site/year for *K. pneumoniae* species complex) and a patient-specific multiplier estimate. b) Prior (grey) and posterior (colored) distributions of patient-specific evolutionary rate multipliers. All posterior distributions are close to those inferred under the main model (Figure 2), suggesting robustness to the coalescent model. Patient identifiers 27.1 and 27.2 correspond to the same patient but different strains, so two evolutionary rates were estimated for this patient.

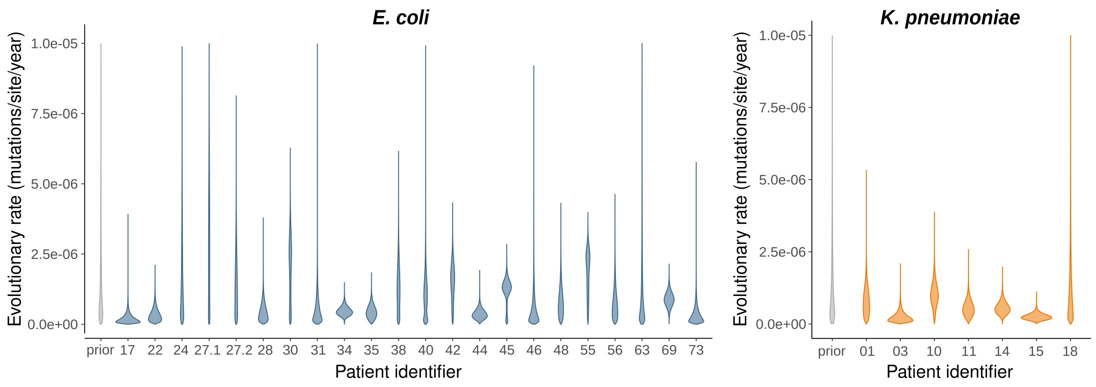

Figure S6: Independent patient-specific within-patient evolutionary rate estimates. Prior (grey) and posterior (colored) distributions of patient-specific within-patient evolutionary rates, estimated for all patients for which at least three serial isolates were available. In contrast to the main analysis, evolutionary rates were estimated independently for each patient. Patient identifiers 27.1 and 27.2 correspond to the same patient but different strains, so two evolutionary rates were estimated for this patient.

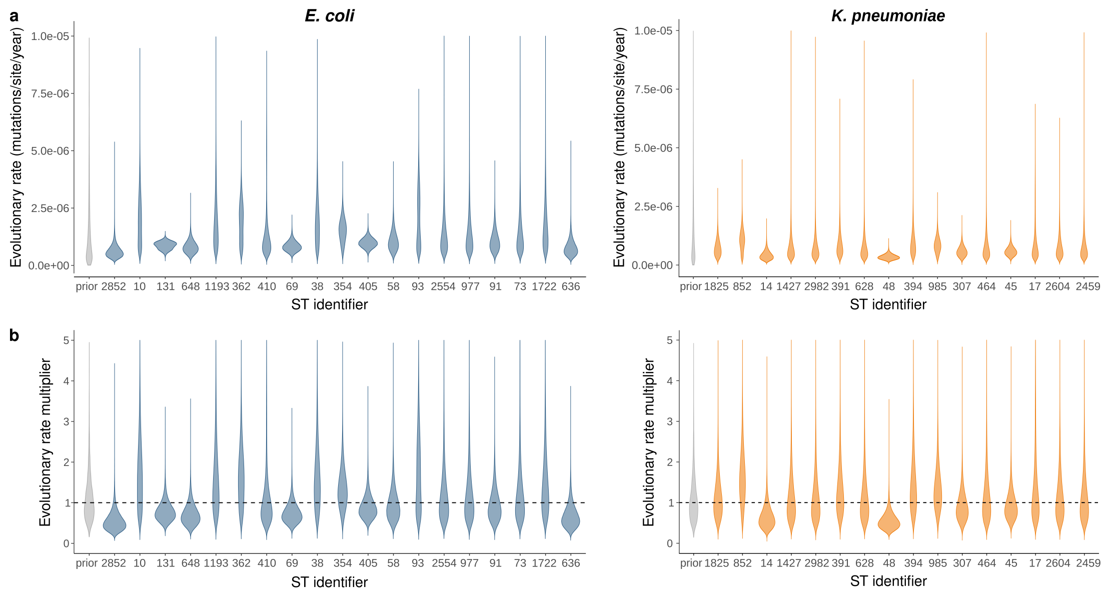

Figure S7: ST-specific within-patient evolutionary rate estimates. a) Prior (grey) and posterior (colored) distributions of ST-specific within-patient evolutionary rates, estimated for all STs on all available isolates. Each ST-specific evolutionary rate estimate corresponds to the product of the average evolutionary rate estimate (1.16e-06 [5.96e-07,1.76e-06] mutations/site/year for *E. coli* and 6.60e-07 [2.99e-07,1.05e-06] mutations/site/year for *K. pneumoniae* species complex) and a ST-specific multiplier estimate. b) Prior (grey) and posterior (colored) distributions of ST-specific evolutionary rate multipliers. The posterior estimates are close to one for most STs, implying that the data do not support an association between ST and evolutionary rate.

References

1. Aguilar-Bultet L, Garcia-Martin AB, Vock I, Maurer Pekerman L, Stadler R, Schindler R, et al. Within-host genetic diversity of extended-spectrum beta-lactamase-producing Enterobacterales in long-term colonized patients. Nat Commun. 2023;14(1):8495. 10.1038/s41467-023-44285-w
2. Wood DE, Lu J, Langmead B. Improved metagenomic analysis with Kraken 2. Genome Biol. 2019;20(1):257. 10.1186/s13059-019-1891-0
3. Parks DH, Imelfort M, Skennerton CT, Hugenholtz P, Tyson GW. CheckM: assessing the quality of microbial genomes recovered from isolates, single cells, and metagenomes. Genome Res. 2015;25(7):1043-55. 10.1101/gr.186072.114
4. Didelot X, Wilson DJ. ClonalFrameML: efficient inference of recombination in whole bacterial genomes. PLoS Comput Biol. 2015;11(2):e1004041. 10.1371/journal.pcbi.1004041
